# CTCF shapes chromatin structure and gene expression in health and disease

**DOI:** 10.15252/embr.202255146

**Published:** 2022-08-22

**Authors:** Bondita Dehingia, Małgorzata Milewska, Marcin Janowski, Aleksandra Pękowska

**Affiliations:** ^1^ Dioscuri Centre for Chromatin Biology and Epigenomics, Nencki Institute of Experimental Biology Polish Academy of Sciences Warsaw Poland

**Keywords:** enhancer, chromatin structure, CTCF, insulator, regulation of gene expression, Chromatin, Transcription & Genomics

## Abstract

CCCTC‐binding factor (CTCF) is an eleven zinc finger (ZF), multivalent transcriptional regulator, that recognizes numerous motifs thanks to the deployment of distinct combinations of its ZFs. The great majority of the ~50,000 genomic locations bound by the CTCF protein in a given cell type is intergenic, and a fraction of these sites overlaps with transcriptional enhancers. Furthermore, a proportion of the regions bound by CTCF intersect genes and promoters. This suggests multiple ways in which CTCF may impact gene expression. At promoters, CTCF can directly affect transcription. At more distal sites, CTCF may orchestrate interactions between regulatory elements and help separate eu‐ and heterochromatic areas in the genome, exerting a chromatin barrier function. In this review, we outline how CTCF contributes to the regulation of the three‐dimensional structure of chromatin and the formation of chromatin domains. We discuss how CTCF binding and architectural functions are regulated. We examine the literature implicating CTCF in controlling gene expression in development and disease both by acting as an insulator and a factor facilitating regulatory elements to efficiently interact with each other in the nuclear space.

## Graphical abstract

This review discusses how CTCF shapes the 3D chromatin structure and chromatin domains, how CTCF binding and architectural functions are regulated, and how CTCF controls gene expression in development and disease by acting as an insulator and a facilitator for regulatory element interaction in the nuclear space.

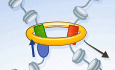

**Glossary**
3C: chromatin conformation capture
3D: three dimensional
AID: auxin inducible degron
CBS: CTCF Bound Site, genomic regions with ChIP‐seq signal indicating CTCF binding
CRE: cis regulatory element
CT: chromosome territory
CTCF: CCCTC‐binding factor
ES cells: embryonic stem cells
FISH: fluorescence *in situ* hybridisation
FRAP: fluorescence recovery after photobleaching
G‐CIMP: CpG island methylator phenotype
GIST: gastrointestinal stromal tumors
Hi‐C: genome‐wide chromosome conformation capture
ICR: imprinting control region
IDH: isocitrate dehydrogenase
IS: insulation score
kb: kilo base pair
Micro‐C: micrococcal nuclease‐assisted chromatin conformation capture
PEI: promoter‐enhancer interactions
PolII: RNA polymerase II
RA: retinoic acid
RBR: RNA‐binding region
RT: residence time
SDH: succinate dehydrogenase
TAD: topologically associating domain
TCGA: The Cancer Genome Atlas
TF: transcription factor
TSS: transcription start site

## Introduction

**Figure 1:**
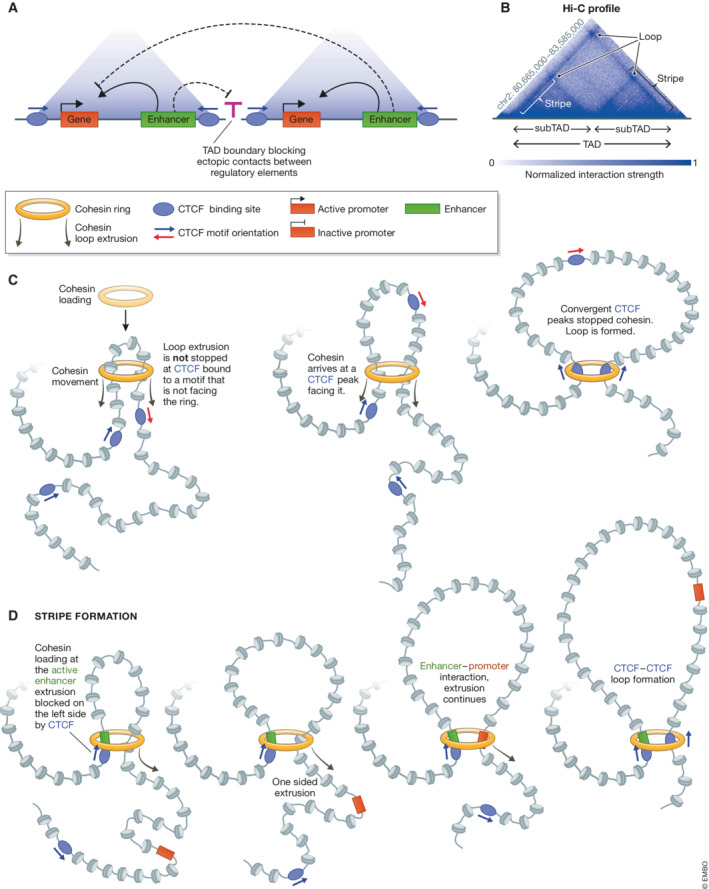
CTCF and cohesins build chromatin architecture (A) Model of Topologically Associating Domains (TAD). TADs are regions of strong self‐contact. Promoter–enhancer interactions inside the domains are favoured while contacts with promoters and enhancers in adjacent domains are restrained. This is believed to help establish a functional organization of the genome. (B) Hi‐C profile illustrating TAD organization at an example locus in Neural Progenitor cells (data from Bonev *et al*, 2017). Increasing colour strength denotes enhanced interaction frequency. This in turn, can be interpreted as increased physical proximity in the three‐dimensional space of the cell nucleus. Triangles of Hi‐C signal reveal domains of enhanced interaction frequencies (TADs). Dots in the matrix (corner peaks) correspond to loops and reveal interactions between relatively short genomic intervals (here sub‐TAD boundaries). At some loci, TAD boundaries interact heavily with the entire TAD, which manifests itself as thin stripes of increased interaction frequency. (C) Loop extrusion model. Upon loading, cohesins (yellow ring) start translocating on chromatin (arrows) and their movement is accompanied by extrusion of an ever‐growing loop. Cohesins pass CTCF proteins bound to a motif which does not face them. Loop extrusion stops when cohesins encounter CTCF bound to a motif that is facing them (thick black arrow). (D) Model explaining the formation of architectural stripes. At genomic locations where cohesin loading occurs in the proximity of CTCF‐binding sites, including at active enhancers (green rectangle), CTCF bound to a motif oriented in a forward direction (*en face*) with respect to the loaded cohesin blocks loop extrusion immediately after loading. Loop extrusion proceeds fuelled by cohesin activity on the other side of the complex and allows the elements in the entire domain including promoters (red rectangle) to be “presented” to the fixed anchor overlapping the active enhancer (green rectangle). Depicted here is a single cohesin ring, it is unclear whether one or two cohesin rings extrude loops.

Cell type‐specific gene expression is ensured by a concerted action of DNA *cis* regulatory elements (CRE) including promoters, enhancers, silencers and insulators. CREs bind transcription factors (TFs) thereby controlling the production of messenger RNAs. Enhancer activity is essential for context‐specific gene expression. Enhancer elements are frequently located at great genomic distances from their cognate promoters and one of the fundamental questions in the field is how, despite pronounced genomic separation, enhancers activate genes with specificity. Several lines of evidence suggest that the way the chromatin fibre is organized in the cell nucleus contributes to ensuring correct promoter–enhancer dialogues. The three‐dimensional organization of chromatin in the cell nucleus is non‐random (Misteli, 2020). Chromosomes occupy distinct territories (CT) and the radial position of a chromosome in the cell nucleus is related to its overall activity. Hi‐C, a genome‐wide chromosome conformation capture technology, allows to look deeply into the organization of the genome highlighting spatial segregation of CTs into A and B compartments that are grossly reminiscent of eu‐ and heterochromatin, respectively (Lieberman‐Aiden *et al*, 2009; Kalhor *et al*, 2011; Rao *et al*, 2014). At genomic distances within a mega base range, which typically separate promoters and enhancers, Hi‐C has revealed that chromatin is arranged into domains of strong self‐contact called topologically associating domains (TADs, Fig 1A and B; Nora *et al*, 2012; Dixon *et al*, 2012; Sexton *et al*, 2012) or contact domains (Rao *et al*, 2014). TADs are intricate in their structures and frequently feature smaller domains referred to as sub‐TADs. High‐resolution fluorescence *in situ* hybridization (FISH), which allows to visualize chromatin structure in 3D space at high resolution, confirmed spatial partitioning of genomes into domains of preferred self‐contact, which correspond to TADs (Wang *et al*, 2016; Bintu *et al*, 2018; Miron *et al*, 2020). FISH revealed that the positions of TAD boundaries vary substantially between cells (Bintu *et al*, 2018). Nonetheless, when averaged across hundreds of alleles, the positions of TAD boundaries inferred from FISH are congruent with the coordinates derived from Hi‐C (Bintu *et al*, 2018; Barth *et al*, 2020). Hence, TAD correspond to individual domains of chromatin organization and Hi‐C can be used to map them.

The observations that: (i) cognate and co‐regulated enhancer–promoter pairs tend to reside in the same TAD (Shen *et al*, 2012; de Laat & Duboule, 2013; Symmons *et al*, 2014), (ii) disruption of TAD boundaries can result in aberrant gene expression of loci in the merged domains (Guo *et al*, 2015; Lupiáñez *et al*, 2015; Franke *et al*, 2016), (iii) genomic intervals under the influence of a regulatory element largely coincide with TADs, as determined by serial insertions of a transcriptional sensor (Akhtar *et al*, 2013; Andrey *et al*, 2017; Despang *et al*, 2019) or of a well‐described promoter–enhancer pair (Zuin *et al*, 2022), and that (iv) co‐regulated enhancers and promoters are located in the same domain (Arner *et al*, 2015), led to the view that through structuring the genome TADs constitute functional units of genome organization (Akhtar *et al*, 2013; de Laat & Duboule, 2013; Kieffer‐Kwon *et al*, 2013; Symmons *et al*, 2014, 2016; Lupiáñez *et al*, 2015; Franke *et al*, 2016; Despang *et al*, 2019). These data together, echoed previous observations that chromatin is organized into functional units in metazoans as was initially appreciated in the fruit fly *Drosophila melanogaster* (Kellum & Schedl, 1991).

In vertebrates, the first chromatin boundaries limiting the action of regulatory elements were discovered at the chicken beta‐globin locus, where these elements either block aberrant gene silencing imposed by proximal heterochromatin (barrier function), or limit enhancer activity (insulator function; Recillas‐Targa *et al*, 1999; Chung *et al*, 1993). Likewise, in the mouse, insulators can shield promoters from being activated by an unrelated enhancer as in the case of the α/δ T‐cell receptor (Zhong & Krangel, 1997) and globin loci (Hanssen *et al*, 2017). Furthermore, insulators orchestrate allele‐specific expression as exemplified at the IGF2/H19‐imprinted gene locus. Deletion of an insulator can lead to inappropriate gene expression and morphological defects in metazoans (Hagstrom *et al*, 1996; Zhou *et al*, 1996; Zhou & Levine, 1999). In essence, the organization of chromatin into functional domains helps to maintain a proper DNA cis‐regulatory element (CRE) dialogue in the cell.

CCCTC‐binding factor (CTCF) is a conserved transcriptional regulator composed of 11 central zinc‐finger domains (ZFs) and peripheral, unstructured N‐ and C termini. CTCF binds a relatively long and complex motif which can be present in the DNA in a forward or reverse orientation. Hence, the motifs of two CTCF bound sites (CBS) can either be in tandem (the same direction), divergent or convergent (facing each other) orientation. As we will see below, the orientation of the motif within a CTCF peak with respect to other genomic features can have profound consequences on chromatin topology and activity (Fig 1).

CTCF was initially uncovered as a protein binding to the chicken Myc promoter, where it associates with a CCCTC‐sequence 180–230 bp upstream of the transcription start site (TSS; Lobanenkov *et al*, 1990; Klenova *et al*, 1993). Around that time, Rainer Renkawitz *et al* described Negative Protein 1 (NeP1), a transcriptional regulator cooperating with nuclear receptors in regulating the chicken lysozyme gene (Baniahmad *et al*, 1990). Later, the authors uncovered that NeP1 is identical to CTCF (Burcin *et al*, 1997). The subsequent discoveries that vertebrate insulators depend on CTCF (Bell *et al*, 1999; Recillas‐Targa *et al*, 2002; Cuddapah *et al*, 2009) and that CTCF contributes to the regulation of the CRE dialogue (Splinter *et al*, 2006; Majumder *et al*, 2008) genuinely transformed the research in the field of transcriptional regulation.

In this review, we outline how CTCF contributes to chromatin architecture. We discuss the function of CTCF as an insulator and recapitulate how CTCF‐bound regions may impact gene expression by integrating and shaping the locus‐specific regulatory landscape in the cell. We summarize recent findings linking CTCF to cell differentiation and disease with a special focus on cancer and neurological disorders.

## CTCF and the cohesin complex build chromatin domains

In mammals, TAD boundaries are enriched in CBS (Dixon *et al*, 2012; Nora *et al*, 2012), which is consistent with the insulator role of CTCF (Bell *et al*, 1999; Recillas‐Targa *et al*, 2002). Cohesin complexes, composed of structural maintenance of chromosomes 1 and 3 and Rad21 (kleisin) and associated factors STAG1/2 and Pds5a/b, form TADs in an energy‐dependent fashion (Gassler *et al*, 2017; Haarhuis *et al*, 2017; Rao *et al*, 2017; Schwarzer *et al*, 2017; Wutz *et al*, 2017; Vian *et al*, 2018). CTCF interacts with cohesins (Rubio *et al*, 2008; Uuskula‐Reimand *et al*, 2016; Li *et al*, 2020), the two factors frequently co‐occupy genomic sites (Parelho *et al*, 2008; Rubio *et al*, 2008; Stedman *et al*, 2008; Wendt *et al*, 2008), and both CTCF and cohesins are required for TAD formation (Sofueva *et al*, 2013; Zuin *et al*, 2014; Nora *et al*, 2017; Wutz *et al*, 2017; preprint: Hsieh *et al*, 2021). Three essential features of TAD structures elucidated our understanding of the mechanisms driving domain and loop formation. First, CTCF‐bound motifs at TAD boundaries are directed primarily toward the interior of the TAD (that is in convergent orientation with respect to the interior of the TAD; de Wit *et al*, 2015; Vietri Rudan *et al*, 2015; Rao *et al*, 2014; Fig 1A). Second, at numerous loci, the two CTCF‐bound domain borders come together to form a loop (Fig 1B), connecting two CBS with convergent motifs (Fig 1A–D; Tang *et al*, 2015; Rao *et al*, 2014). Third, the fact that a relatively small protein (CTCF is ~5 nM in diameter) dictates formation of a large chromatin structure (TADs and loops are several hundreds of nanometres wide) in a way that is dependent on the orientation of its motif, collectively hinted at a one‐dimensional (1D) loop extrusion as the most likely mechanism underlying TAD formation (Nasmyth, 2001; Alipour & Marko, 2012; Dekker & Mirny, 2016). Computational simulations and experimental assessment of chromatin folding in cells with genetically engineered perturbations of CTCF motifs supported the loop extrusion hypothesis (Sanborn *et al*, 2015; Fudenberg *et al*, 2015; Guo *et al*, 2015; Fig 1C and D). In the loop extrusion model, upon loading, cohesins slide along the chromatin fibre and extrude a loop. This activity is stopped when they encounter CTCF that is bound to a motif that faces them. Recently, dedicated microfluidics devices coupled with fluorescence imaging, allowed for the direct visualization of cohesin‐mediated loop extrusion *in‐vitro* and in real‐time (Davidson *et al*, 2019; Kim *et al*, 2019; Golfier *et al*, 2020). It will be an exciting and ground‐breaking development to track chromatin loop extrusion in the living cell nucleus in real time.

## CTCF can act as insulator and chromatin barrier element

**Figure 2:**
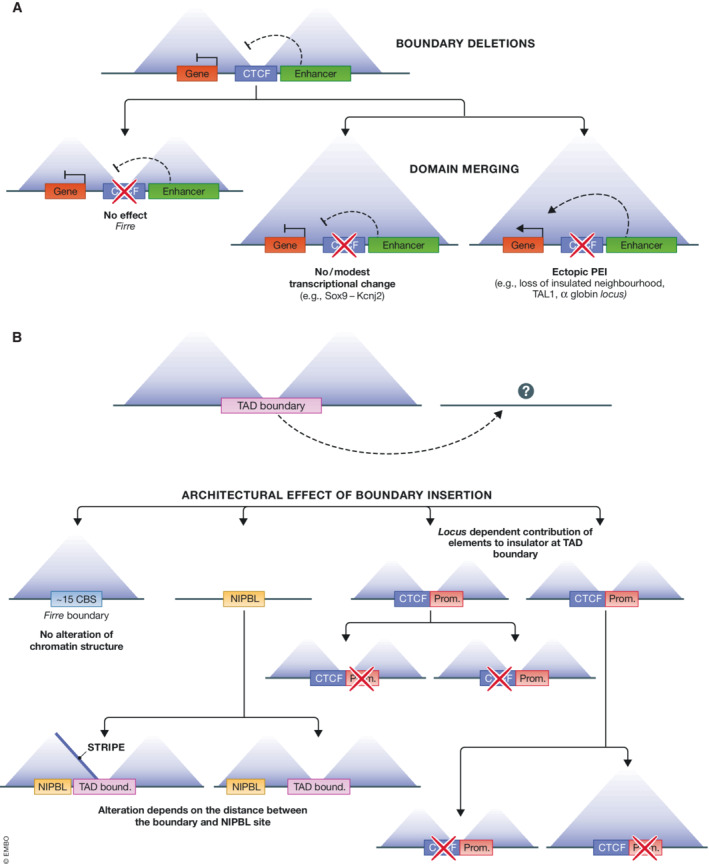
Genome engineering reveals locus‐specific transcriptional and architectural consequences of TAD boundary deletion and insertion (A) TAD or a sub‐TAD boundary deletion may lead to no overt alteration of chromatin architecture as seen at the Firre locus (Barutcu *et al*, 2018), or to TAD and subTAD merging accompanied by either modest (Sox9/Kcjn2; Despang *et al*, 2019) or considerable transcriptional changes (e.g., loss of insulated neighbourhoods and oncogene activation; Hnisz *et al*, 2016), or aberrant activation of genes as, for example in the vicinity of otherwise insulated globin genes (Hanssen *et al*, 2017). (B) Ectopic insertion of a boundary element may lead to no change in the architecture of the recipient locus (Barutcu *et al*, 2018). When considering other CBS, boundaries can still be formed despite the deletion of the CBS. Depending on whether the ectopic boundary is inserted far or close to a Nipbl cohesin loader binding site, the boundary may form stripes (Redolfi *et al*, 2019). The contribution of distinct elements making up the boundary depends on the intrinsic features of the target locus. At one location, a boundary composed of a CBS site and a housekeeping gene promoter depends on both elements, while at another location CTCF appears less crucial for boundary formation (Zhang *et al*, 2020).

Loss of CTCF binding at TAD boundaries can perturb their capacity to insulate contacts between domains leading to merging of adjacent TADs (Despang *et al*, 2019; Franke *et al*, 2016; Lupiáñez *et al*, 2015; Guo *et al*, 2015; Hanssen *et al*, 2017; Vian *et al*, 2018; Fig 2A). Functionally, TAD boundary deletion causes a spectrum of effects ranging from minor, as at the *Sox9‐Kcnj2* locus (Despang *et al*, 2019) to substantial. For instance, deletion or inversion of TAD boundaries at the *WNT6*‐*EPHA4*‐*PAX3* locus may lead to alterations in mouse digit number (Lupiáñez *et al*, 2015). At the murine alpha globin locus, removal of CBS that partition the domain into smaller sub‐TADs, leads to aberrant upregulation of genes otherwise silenced in the presence of the sub‐TAD boundary (Hansen *et al*, 2019). Essential cell identity genes are often demarcated by CTCF/cohesin loop anchors. Loop formation insulates these genes together with their regulatory neighbourhood, which helps to maintain the local chromatin environment and proper gene expression. Removal of the anchors of such loops leads to misexpression of key regulators of cell fate (Dowen *et al*, 2014) and can contribute to diseases including cancer (as will be discussed below).

The auxin‐inducible degradation (AID) system allows to deplete a protein of interest efficiently and rapidly (Natsume *et al*, 2016). AID‐mediated acute removal of CTCF weakens TAD boundaries and disrupts CTCF–CTCF loops (Nora *et al*, 2017; Wutz *et al*, 2017; Hyle *et al*, 2019), establishing an essential role of CTCF in the formation of TADs. (It is worth noting here that despite a general effect, some borders are insensitive to the depletion of CTCF protein hinting at additional mechanisms driving the segmentation of the genome.) Acute removal of CTCF leads to transcriptional deregulation of numerous loci in embryonic stem (ES) cells, immortalized erythroid precursors, and B cell cancer cells (Nora *et al*, 2017; Hyle *et al*, 2019; Xu *et al*, 2021). Even in short time scales, loss of functional insulators could lead to global gene deregulation. The acute depletion of CTCF results in equal numbers of up and downregulated genes. Gene upregulation in the CTCF‐depleted cells can, to some degree, be explained by loss of boundary activity. In comparison with genes not affected by CTCF loss, loci with gained expression in the CTCF‐depleted cells are more frequently separated from nearby enhancers by a TAD boundary (Nora *et al*, 2017). Yet, despite prominent examples of gene deregulation in the absence of CTCF, the direct transcriptional effects of CTCF removal remain overall mild, as one would predict massive transcriptional changes upon genome‐wide abrogation of insulator activity (preprint: Hsieh *et al*, 2021; Luan *et al*, 2021). The lack of stronger effects may be due to only a limited dependence of gene expression on long‐range enhancer regulation. Likewise, epigenetic silencing of promoters could render them unresponsive to enhancers. Furthermore, promoter–enhancer specificity could be more often hardwired at the level of biochemical compatibility between CREs (Pachano *et al*, 2021) versus being regulated by insulators. Finally, a recent study revealed hundreds of promoter–enhancer interactions (PEI) that cross TAD boundaries suggesting that multiple enhancer–promoter pairs are perhaps unaffected by the insulating function of CTCF (preprint: Hsieh *et al*, 2021; see also below). Interestingly, prolonged depletion of CTCF leads to substantial gene deregulation (Nora *et al*, 2017) and is incompatible with cell differentiation (see below). Together, these results indicate that the functional perturbations elicited by the loss of CTCF are either caused by secondary effects subsequently to the initial deregulation of a handful of genes (Hyle *et al*, 2019; Xu *et al*, 2021), or that the direct effects of CTCF removal require time to unfold (preprint: Hsieh *et al*, 2021).

Chromatin boundaries may act as barrier elements that block spreading of heterochromatin, thereby inhibiting gene silencing. In essence, barrier activity allows to overcome chromatin position effects and shield genes to ensure stable expression despite repressive chromatin environments. Several paradigm insulators including 5'HS4 element at the chicken beta‐globin locus feature both insulator and barrier activities (Recillas‐Targa *et al*, 2002). Genome wide, CBS frequently coincide with zones of transition between open chromatin and histone 3 lysine 27 trimethylation (H3K27me3)‐enriched domains (Cuddapah *et al*, 2009). However, acute removal of CTCF in ES cells does not lead to spreading of H3K27me3 to adjacent domains (Nora *et al*, 2017), consistent with previous reports revealing uncoupling between CTCF and of 5'HS4 barrier activity at the chicken beta‐globin locus (Recillas‐Targa *et al*, 2002). Can CTCF nonetheless segregate regions of diverse chromatin activity? Hox gene clusters constitute paradigm loci for studying the interplay between trithorax and polycomb group proteins. The two complexes establish euchromatic H3K4me3 and heterochromatic H3K27me3 histone marks, respectively, thereby regulating spatiotemporal patterns of Hox gene expression, which are essential for axial patterning of the developing embryo. Exposure of ES cells to retinoic acid (RA) mimics cervical motor neuron development. At the *HoxA* locus, RA treatment of ES cells leads to upregulation of *HoxA1‐6* rostral genes, leaving *HoxA7‐13* silent (consistent with the pattern of expression during embryogenesis, *HoxA7‐13* are normally expressed in caudal parts of the embryo). The boundary between *HoxA1‐6* and *HoxA7‐13* genes associates with CTCF; the removal of this CBS leads to spreading of histone marks related to open chromatin, loss of H3K27me3 and transcriptional activation of the *HoxA7* gene (Narendra *et al*, 2015). The loss of the barrier role of CTCF is translated into gain of regulatory interactions at the *HoxA* locus and homeotic transformations of the embryo (Narendra *et al*, 2016).

Together, these experiments show essential roles for CTCF in the formation of TADs. TADs overall help maintain proper CRE dialogues in the cell. Through CTCF, TAD boundaries act as insulators and at some genomic locations as barrier elements. Yet, the contribution of TAD boundaries appears locus and context‐specific. It will be important to determine which CBS constitute genuine insulators and what properties of the domains and TAD sequences allow some CBS to exert enhancer‐blocking functions (Box: In need of answers). Likewise, it will be essential to determine how many barrier elements there are in the mammalian genome, and how they depend on CTCF.

**Box: In need of answers**

i What mechanisms determine the choice of CBS for boundary function?
ii How does RNA contribute to CTCF–CTCF loop formation and boundary activity, and can RNA affect the stability of CTCF dimers at the anchor of architectural loops?
iii What mechanisms underlie the genome‐wide maturation of chromatin topology during embryonic stem cell differentiation?
iv How does the local chromatin environment impact insulator activity and how do promoter‐enhancer interactions depend on CTCF?
v How do post‐translational modifications and protein partners of CTCF contribute to its architectural functions?

## Harnessing the unique properties of CTCF at gene promoters and enhancers

CBS frequently overlap promoters (Chen *et al*, 2012). Historically, the functions of CTCF were addressed at the P2 element, a CBS immediately downstream of the transcription start site (TSS) of *Myc*. The impact of CTCF at P2 is largely repressive (Filippova *et al*, 1996). Similarly, CBS within the silencer of the chicken lysozyme gene (LYZ; Arnold *et al*, 1996; Burcin *et al*, 1997), as well as CBS adjacent to thyroid hormone responsive element in the 3'UTR of the LYZ gene or the intronic CBS at the HLA‐DRB1 locus were suggested to inhibit gene expression based on *in‐vitro* reporter assays (Awad *et al*, 1999; Arnold *et al*, 2000). Can CTCF repress gene expression, and can it do so directly, by binding to promoters? It is hard to answer this question without an extensive effort in genome engineering, but acute CTCF depletion experiments may provide some insight into this matter.

Transcriptional activation corollary to acute CTCF loss in B cell leukemic cells is linked to gain in chromatin accessibility at promoters (Xu *et al*, 2021). This result is compatible with CTCF acting directly at the promoter to inhibit gene expression. CTCF can induce DNA bending (Arnold *et al*, 1996) which might impact binding of other proteins to DNA thereby silencing gene expression. Furthermore, CTCF can interact with a co‐repressor SIN3A, which can remodel chromatin and induce histone deacetylation leading to reporter gene downregulation (Lutz *et al*, 2000).

CTCF can also exert a stimulatory role when bound to gene promoters and the mechanisms of CTCF‐mediated positive impact on transcription might entail the regulation of both the local chromatin landscape and the large‐scale genome architecture. CTCF can co‐purify with the largest subunit of RNA Polymerase II (PolII; Chernukhin *et al*, 2007), promote PolII clustering in the nucleus (Lee *et al*, 2022) and recruit TBP‐associated core promoter factor TAF3 to upregulate gene expression (Liu *et al*, 2011). Diminished levels of CTCF lead to loss of a fraction of peaks of CTCF at promoters and transcriptional downregulation of genes implicated in oncogenesis (Aitken *et al*, 2018; see also below). Promoters of genes that are downregulated upon removal of CTCF frequently contain CBS and, at a subset of these promoters, CTCF binds only 60 bp upstream of the TSS. At these sites, the CTCF motif is predominantly oriented in the same direction as the gene promoter (Nora *et al*, 2017). The significance of this observation is unclear. Given that CTCF binding is related to well‐positioned nucleosome arrays, CTCF might impact gene expression by orchestrating nucleosome phasing (Nora *et al*, 2017). Alternatively, by limiting antisense transcription, CTCF might favour gene expression (Cho *et al*, 2005; Degner *et al*, 2011). Why only a small minority of CTCF‐bound promoters (10%, Nora *et al*, 2017) cause transcriptional upregulation in response to CTCF depletion remains unclear.

The positive role of CTCF at gene promoters likely relies, at least in part, on its architectural functions. Promoter–enhancer loops can connect convergently oriented CBS with distal CREs including enhancers and promoters (Rao *et al*, 2014; Tang *et al*, 2015). Hence, in addition to impacting nucleosome positioning, CBS within gene promoters might act as anchor or docking sites that facilitate PEI. For instance, a CBS located 2 kb upstream of the MYC promoter is conserved in multiple cancer cell lines. This CBS is required for the MYC promoter to receive input from various cell type‐specific super‐enhancers located in the genomic surroundings of the *MYC* gene (Schuijers *et al*, 2018). Many more genes harbour CTCF sites in their extended promoter region that could potentially exert enhancer docking functions (Schuijers *et al*, 2018) and modify the affinity of promoters to enhancers (Oh *et al*, 2021b). The extent to which this model describes the implication of CTCF in regulation of oncogene expression is, however, under debate (Hyle *et al*, 2019).

Promoters may display enhancer activity towards other genes (Dao *et al*, 2017; Diao *et al*, 2017). Artificial tethering of CTCF to the *Vcan* promoter facilitates its transcriptional upregulation during neural induction via establishing contacts with the promoter of the *Tmem167* gene located 350 kb downstream of the *Vcan* TSS (Kubo *et al*, 2021). Likewise, CBS in the vicinity of enhancers may act to favour the interaction with their cognate promoter. At the human Sonic hedgehog (*SHH*) locus, CTCF sites flanking the limb‐specific enhancer ZRS are required for *SHH* expression; lack of these CBS is related to acheiropody, a congenital condition featuring limb truncation (Ushiki *et al*, 2021).

The mechanism of CTCF‐driven formation of PEI most likely depends on cohesin action. The cohesin loader Nipbl is enriched at active enhancers (Kagey *et al*, 2010; Kieffer‐Kwon *et al*, 2013; Liu *et al*, 2021) and proper cohesin dynamics are essential for promoter–enhancer transactions. Loss of cohesins diminishes PEIs (Lavagnolli *et al*, 2015; el Khattabi *et al*, 2019) affecting the capacity of enhancers to fully upregulate genes (Aljahani *et al*, 2022). Removal of cohesins primarily affects genes regulated by remote enhancers (Lavagnolli *et al*, 2015; Calderon *et al*, 2022). Likewise, failure to remove cohesin complexes from chromatin also affects PEI. Deletion of the Wapl cohesin unloader leads to exhaustion of the free cohesin pool in the nucleoplasm thereby blocking loading of new cohesin complexes at enhancers affecting timely formation of PEIs (Liu *et al*, 2021). At genomic sites where cohesins are loaded in close vicinity of CBS with a motif oriented *en face* of the newly loaded loop extruders, cohesin activity is immediately blocked on one site which leads to fixation of the cohesin loading enhancer at the anchor of the loop (Fig 1D). In this configuration, extrusion proceeds on the other side of the ring reeling in and presenting the entire TAD to the enhancer, in Hi‐C maps this is accompanied by formation of architectural stripes (Barrington *et al*, 2019; Vian *et al*, 2018; Fig 1B and D). Recently, using an orthogonal, crosslinking‐free method to score for chromatin structure, the Giorgetti lab has provided evidence that cohesin loading in the vicinity of a CBS leads to the formation of stripes (Redolfi *et al*, 2019).

CTCF removal leads to loss of numerous PEI (Thiecke *et al*, 2020; Kubo *et al*, 2021; Lee *et al*, 2022) and a reversible disruption of PolII‐enriched transcriptional condensates (Lee *et al*, 2022). Yet, as we have seen above, the immediate transcriptional consequences remain somewhat scarce and the acute degradation of CTCF does not lead to overt changes in transcription initiation or elongation genome‐wide (preprint: Hsieh *et al*, 2021; Luan *et al*, 2021). Likewise, although substantial data point to an essential role of TADs in the spatiotemporal regulation of gene expression (de Laat & Duboule, 2013), the relationship between the three‐dimensional structure of chromatin, TAD formation and transcriptional regulation appears complex. While activation of genes frequently coincides with formation of promoter–enhancer loops as detected by live cell microscopy (Chen *et al*, 2018), chromatin conformation capture assays (Sanyal *et al*, 2012; Kieffer‐Kwon *et al*, 2013; Mifsud *et al*, 2015; Bonev *et al*, 2017; Pekowska *et al*, 2018; Hua *et al*, 2021) and genome architecture mapping (Beagrie *et al*, 2017), these techniques show that promoter–enhancer loops can also be formed in the absence of enhancer activity (de Laat & Duboule, 2013; Ghavi‐Helm *et al*, 2014; Williamson *et al*, 2016; Phanstiel *et al*, 2017). At some loci, the distance between promoters and their cognate enhancers can be uncoupled from gene activation (Alexander *et al*, 2019; Benabdallah *et al*, 2019). Promoters might form only a transient interaction with condensates containing the mediator complex (Cho *et al*, 2018b). Such an extremely dynamic nature of PEI would hence require an ultra‐deep sequencing of Hi‐C libraries for detection (Bonev *et al*, 2017; Hua *et al*, 2021). Technological improvements, including the introduction of micro‐C which allows to detect substantially larger repertoires of PEIs due to a higher signal to noise ratio (preprint: Hsieh *et al*, 2021), indicate that PEI rely on physical contacts. Piggy‐Back‐mediated genetic engineering experiments allowed to place an enhancer at increasing genomic distances from its cognate promoter and, combined with Hi‐C, recently demonstrated a complex interplay between physical proximity, presence of other CREs in a TAD and functional interactions between regulatory elements stressing the need for context‐specific models of PEI control (Zuin *et al*, 2022).

While the data suggest that CTCF is implicated in the setup of PEI, recent reports show that a substantial fraction of PEIs appears immune to acute (3 h) removal of CTCF or cohesins, suggesting a “time‐buffering” model, where the established local chromatin environment would be sufficient to keep PEI for at least 3 h without these architectural factors (preprint: Hsieh *et al*, 2021). Micro‐C also revealed thousands of previously unappreciated PEIs. Remarkably, in mouse embryonic stem cells, 20% of the PEIs connect elements located in two adjacent TADs, and are hence not blocked by CTCF‐bound borders (preprint: Hsieh *et al*, 2021). This discovery is interesting in light of the observation that, in the context of an engineered locus, where a CTCF site is located between an enhancer and its cognate promoter, truncations in the enhancer element that diminish its strength (as established by reporter assays) also render the action of the enhancer more susceptible to insulation by CTCF (Zuin *et al*, 2022). It will be fascinating to assess more broadly whether inter‐TAD PEIs are functional and how the enhancer strength contributes to the capacity of PEIs to overcome the constrains imposed by CTCF.

Deletion of CTCF sites leads to increased heterogeneity of gene expression mouse T cells (Ren *et al*, 2017). Likewise, CTCF and cohesin depletion cripples the response of macrophages to inflammatory stimuli (Cuartero *et al*, 2018; Stik *et al*, 2020) and can reduce the impact of enhancers on a subset of promoters (Vos *et al*, 2021; Aljahani *et al*, 2022) perhaps by influencing the formation of transcriptional condensates (Lee *et al*, 2022) or by regulating the capacity of TFs to bind to chromatin (preprint: Hsieh *et al*, 2021). These data indicate that rather than strictly allowing, CTCF modulates PEIs. It remains an open question how PEIs are formed and how the specificity of these interactions is ensured. We predict that further technological developments will reveal an even larger complexity of PEIs, and a combination of live‐cell imaging and improved chromatin conformation capture assays will most likely be instrumental to address these questions (Brandão *et al*, 2021). (See also Concluding remarks.)

## Regulatory element composition and genomic context impact strength of insulators

Insulation score (IS) is defined as the ratio between the number of interactions that cross a given genomic position and the number of interactions that are formed at both sides of the assayed position (Sofueva *et al*, 2013). Using advanced computational methods to increase the robustness of Hi‐C, allowed Gong *et al* to classify TAD boundaries based on their IS. Apart from showing that the strongest boundaries tend to have more pronounced CTCF binding (Gong *et al*, 2018), this analysis revealed that the cell‐type invariant TAD boundaries differ in strength between tissues (Gong *et al*, 2018) as also shown in developing cells (Pekowska *et al*, 2018). Hence, the IS constitutes a tuneable parameter of chromatin architecture.

The IS reflects the fraction of interactions built on both sides of a boundary to the ones that cross it. Cohesin loading and hence loop extrusion is not uniform across the genome (Vian *et al*, 2018; Hua *et al*, 2021) and it is unclear how the density of the loop‐extruding complexes impacts the IS. Recent data show that both boundary‐encoded, and locus intrinsic features shape the capacity of CBS to sustain insulator functions (Fig 2). When inserted into an unrelated genomic locus, both an artificial construct containing three CBS and a TAD boundary separating the *HoxD* locus into two domains retain their insulator functions at the ectopic site (Redolfi *et al*, 2019; Willemin *et al*, 2021). In contrast, the CTCF‐enriched TAD boundary intersecting the long‐noncoding RNA locus *Firre*, exerts no overt effect on chromatin structure at an ectopic locus (Barutcu *et al*, 2018). Hence, the capacity of the TAD boundaries to insulate regions from each other in some cases depends on the site where they are inserted.

TAD boundaries frequently coincide with CTCF‐binding sites and housekeeping gene promoters (Dixon *et al*, 2012). To what extent do these elements collaborate to regulate the IS? A boundary element containing both the CTCF‐binding site, and the transcription start site (TSS) of a housekeeping gene *PARL*, requires both elements to exert its function (Zhang *et al*, 2020). Yet, the strength of insulation depends not only on the CBS and *PARL* promoter but also on the local chromatin context of the region where the boundary was knocked in. Removal of the CBS in the inserted boundary reduced the IS; while the excision of the TSS from the boundary exerted a more pronounced effect on IS at one ectopic locus. In contrast, when inserted at another genomic position, the TSS and CBS of the same boundary worked in an additive fashion (Zhang *et al*, 2020). The presence of the CBS within the ectopic locus and the distance of the inserted boundary to the nearest transcribed gene contribute to the IS of the boundary at the insertion site (Zhang *et al*, 2020). Thus, is transcription sufficient to elicit boundary formation? In the absence of CTCF, cohesins accumulate at sites of convergent transcription which might in principle lead to enhanced contact insulation and boundary formation (Busslinger *et al*, 2017). When inserted into the coding sequence of a gene, the TSS of *PARL* gene alone can drive boundary formation (Zhang *et al*, 2020). The fact that a fraction of boundaries gained upon ES cell differentiation is devoid of CTCF (Pekowska *et al*, 2018), but overlaps activated promoters, as exemplified at the *Zfp608* locus (Bonev *et al*, 2017), suggests an instructive role of promoters in chromatin organization during differentiation. Yet, a precocious transcriptional activation of the *Zfp608* promoter by dCas9‐VP64 is insufficient to elicit insulation (Bonev *et al*, 2017), which means that additional factors recruited to the *Zfp608* promoter during differentiation might be required for boundary formation. The inhibition of transcription can lead to diminished boundary strength (Rowley *et al*, 2017; Barutcu *et al*, 2019) but the effect appears relatively weak and numerous other reports show no impact of a transcriptional block on TAD structures in the fruit fly (Hug *et al*, 2017; Hsieh *et al*, 2020; Jiang *et al*, 2020). A transcriptional block does not affect the restoration of chromatin loops neither upon reintroduction of cohesins (Vian *et al*, 2018) nor upon entry into the G1 phase after cell division (Zhang *et al*, 2021). Interestingly, emergence of TAD boundaries in fruit fly oocytes depends on the TF Zelda that induces a global transcriptional onset (Hug *et al*, 2017). It will be important to define which factors regulate CTCF‐less boundaries in mammalian cells and whether these factors might also impact CTCF‐bound insulators.

Using a highly efficient flippase‐assisted recombination‐based genome engineering, Huang *et al* assessed a set of CTCF‐bound TAD boundary elements for their insulator activity by inserting them between the Sox2 promoter and a super‐enhancer active in mouse ES cells (Huang *et al*, 2021). Insulators exerted a remarkably weak effect on Sox2 expression, out of the 11 tested CBS, the most potent element reduced the expression of Sox2 by only 11%. Inclusion of an increasing number of tandemly oriented CBS enhanced the IS and diminished the activity of Sox2 gene by at most ~40%, and the transcriptional effect correlated with the extent of changes in chromatin structure measured by Hi‐C (Huang *et al*, 2021). Does this mean that insulators exert only a very modest impact on gene expression? Or rather, that insulator functions are a derivative of both sequence composition of the boundary and the local chromatin environment as shown by Barutcu *et al* (2018)? Sleeping‐beauty transposon‐assisted insertion of a fluorescent reporter construct containing a strong enhancer and a weak promoter separated by a well‐established insulator revealed that reporter activity strongly depends on the presence of additional CREs at the locus (Ribeiro‐Dos‐Santos *et al*, 2022). Likewise, older *in‐vivo* experiments in the fruit fly show that the strength of the Fab‐7 boundary element depends on the promoter–enhancer pair, and some enhancers are blocked by insulators more readily than others (Zhou *et al*, 1996). A similar observation was recently made when assessing the capacity of a CBS to interfere with PEI at an engineered locus (Zuin *et al*, 2022). Taken together, these data indicate that transcriptional output at a given locus is most likely corollary to the combined action of multiple elements that dynamically interact and signal to one another thereby collectively moulding the activity of the locus. This property of transcriptional regulatory systems likely underlies the difficulty to assess insulator functions of CTCF sites in chromatin reporter assays that largely rely on insertion of insulator elements in‐between a known enhancer–promoter pair. The low activity of insulators in genomic engineering experiments might in part be due to the disability of ectopic sites chosen for the assay to provide the necessary environment for proper insulator action. Large scale *in‐situ* assessments of the implications of CTCF sites for regulating promoter–enhancer transactions will be needed to critically assess this model.

## Intrinsic and extrinsic factors regulate CTCF binding to its cognate sites

CTCF interacts with an array of motifs with marked differences in DNA sequence. It does so by deploying distinct combinations of its ZFs depending on the site (Filippova *et al*, 1996, 1998; Burcin *et al*, 1997; Awad *et al*, 1999; Kanduri *et al*, 2000; Quitschke *et al*, 2000; Renda *et al*, 2007). The ZFs of CTCF interact with multiple DNA bases simultaneously, ZFs 4‐7 bind to the major groove of DNA, the interaction involves only one strand of the DNA double‐helix (Hashimoto *et al*, 2017; Yin *et al*, 2017). By overexpressing ZF mutants of CTCF and using ChIP‐seq to localize the engineered CTCF molecules, Nakahashi *et al* showed that not all the ZFs contribute equally to CTCF DNA binding (Nakahashi *et al*, 2013). ZF 4‐7 appear essential for the pattern of CTCF distribution, their mutants occupy less than 20% of the wild‐type CBS. These central ZF were previously annotated as binding to the core DNA‐binding motif (Filippova *et al*, 1996; Renda *et al*, 2007; Ohlsson *et al*, 2010). Recently, combining the acute depletion of the wild‐type form of CTCF with controlled expression of ZF CTCF mutants, allowed Soochit *et al* to obtain largely improved conditions to study the impact of individual ZFs; the ZFs mutants that destabilize CTCF binding to the largest degree display highest CTCF‐DNA‐binding dynamics and lowest capacity to form loops (Soochit *et al*, 2021). Therefore, the stability of the association between CTCF and DNA can be linked to loop formation and the control of chromatin architecture. Residence time (RT) of CTCF on chromatin measured by Fluorescence Recovery After Photobleaching (FRAP) is within a range of minutes in mouse ES cells (Hansen *et al*, 2017). This value appears high when compared to more classical transcriptional regulators. Given the link between the RT of CTCF binding and loop formation (Soochit *et al*, 2021), it is possible that a long RT is a perquisite for CTCF's architectural functions. Live‐cell imaging and tracking of CTCF‐CTCF loop anchors recently revealed that a loop at the *Fbn2* locus can persist for up to 30 minutes in ES cells (Gabriele *et al*, 2022). Loop stability might be even more pronounced; estimates based on the measurements of the stability of cohesin binding to chromatin indicate that loop structures may persist for hours depending on the post‐translational modifications of STAG factors (Wutz *et al*, 2020). Yet, it remains unclear and relatively understudied whether RT of CTCF differs between tissues and whether chromatin loop formation can be impacted by mechanisms that influence the RT of CTCF in physiological settings. Remarkably, activation of quiescent B cells by exposure to conditions mimicking immune responses leads to a marked reduction of the RT of CTCF (Kieffer‐Kwon *et al*, 2017). In the future, it will be instrumental to understand the extent by which the RT of CTCF differs between cell types and how it is related to its architectural functions.

### DNA methylation anticorrelates with CTCF binding and insulator activity

DNA methylation anticorrelates with CTCF binding (Bell & Felsenfeld, 2000); differences in the methylation of CpGs islands correlate with cell type‐specific CTCF binding and insulator activity (Wang *et al*, 2012). The imprinting control region (ICR) that regulates allele‐specific expression of Igf2 and H19 binds to CTCF; the insulator function of ICR depends on DNA methylation that anti‐correlates with insulation (Bell & Felsenfeld, 2000; Hark *et al*, 2000; Kanduri *et al*, 2000; Szabó *et al*, 2000; Cui *et al*, 2001; Holmgren *et al*, 2001). An analogous situation has been described at the Gtl2 and Dlk1 loci, where the ICR is hemi‐methylated and binds to CTCF at the unmethylated allele (Wylie *et al*, 2000). CTCF binding at other imprinted loci (Hikichi *et al*, 2003; Fitzpatrick *et al*, 2007; Lin *et al*, 2011) including *Rasgrf1*, *Myotonic Dystrophy 1 (MD1)*, is also sensitive to DNA methylation (Filippova *et al*, 2001; Yoon *et al*, 2005). Mutations in isocitrate dehydrogenase (IDH) or succinate dehydrogenas (SDH) cause DNA hyper‐methylation in glioblastomas and gastrointestinal stromal tumors (GIST), respectively. The increased DNA methylation affects CTCF binding and insulator function, which favours oncogene expression (Flavahan *et al*, 2016, 2019). While KIT‐mutant, PDGFRA‐mutant and SDH‐mutant GIST share enhancer landscapes, they differ in transcriptional programmes. The CBS hyper‐methylation and insulator dysfunction in SDH‐mutant GISTs largely explains the differences in gene expression and in the future may help to contribute to the development of personalized anti‐cancer therapies (Flavahan *et al*, 2019).

However, while unmethylated motifs are bound by CTCF preferentially (Stadler *et al*, 2011; Feldmann *et al*, 2013), DNA methylation does not block the association between CTCF and DNA *in vivo* (Stadler *et al*, 2011). Likewise, only 40% of tissue or cell type‐specific CBS can be related to differential DNA methylation and genome‐wide loss of DNA methylation does not lead to a massive unmasking of CTCF motifs and a marked gain in new CBS (Stadler *et al*, 2011; Wang *et al*, 2012). While DNA demethylation exerts overall weak effects on the profile of CTCF binding, induction of DNA methylation seems to have a more pronounced effect. Genetic removal of ten‐eleven translocation 1 and 2 (Tet1 and Tet2) dioxygenases, that convert 5‐methylcytosine into hydroxymethylated, formylated (5fC) or carboxylated intermediates, increases DNA methylation and causes the loss of a substantial fraction of CTCF peaks in ES cells (3,916 CBS were lost, while 7,232 CBS were maintained in the Tet1/2^−/−^ cells). This effect, pronounced at regions with low CpG density, is possibly caused by nucleosome repositioning and occlusion of CTCF motifs rendering them inaccessible to CTCF (Wiehle *et al*, 2019). In general, sites with a low CpG content seem to bind CTCF less and appear particularly vulnerable to DNA methylation levels (Wiehle *et al*, 2019). In ES cells, CRISPR‐dCas9‐Dnmt3a‐mediated methylation of the CTCF‐binding site insulating *Nlrp12* and *H2Q10* loci from the expressed *miR290* and *Pou5f1* genes caused transcriptional upregulation of *Nlrp12* and *H2Q10* (Liu *et al*, 2016). However, it needs to be determined whether the effect is caused by DNA methylation or by a possible occlusion of the CBS by the dCas9 protein.

### Histone modifications and chromatin openness impact CTCF binding

CBS were originally annotated by analysing DNAseI sensitive sites. CTCF‐binding motifs are depleted of nucleosomes (Teif *et al*, 2012; Carone *et al*, 2014; Liu *et al*, 2016) and CBS feature up to 20 well‐positioned nucleosomes around the CTCF motif (Fu *et al*, 2008). Chromatin openness could be one of the signatures tagging CTCF motifs for recognition. Yet, open regions that intersect CTCF peaks are closed upon CTCF removal (Xie *et al*, 2020). CTCF interacts with chromatin remodelling complexes including switch/sucrose nonfermentable complex (SWI/SNF) and the Imitation SWItch (ISWI) complex (Wiechens *et al*, 2016; Marino *et al*, 2019; Valletta *et al*, 2020). The removal of *Snf2h*, the ATPase subunit of the ISWI complex reduces CTCF chromatin binding and CTCF‐CTCF loops in ES cells (Wiechens *et al*, 2016; Barisic *et al*, 2019). The latter result is somewhat unexpected, as the deletion of *Snf2h* leads to a *reduction* not abrogation of CTCF binding (Barisic *et al*, 2019), and according to the targeted protein degradation experiments, removal of over 90% of CTCF is required to detect robust loop loss in ES cells (Nora *et al*, 2017). The more recent data suggest that even relatively subtle changes in strength of CTCF binding (as detected by ChIP‐seq) may translate to pronounced architectural effects. It will be interesting to assess how Snf2h loss impacts the dynamics of CTCF binding to chromatin in real time. CTCF can promote chromatin opening and incorporation of a histone variant H3.3 (Weth *et al*, 2014) suggesting that it can act upstream of the establishment of DNA accessibility. H2A.Z, a histone variant of H2A, promotes nucleosome unwrapping, and surprisingly, the removal of H2A.Z can enhance CTCF binding (Wen *et al*, 2020), suggesting a destabilizing role for these euchromatin‐enriched histone variants in CTCF binding.

### CTCF‐binding sites implicated in chromatin topology are ultra‐stable

Acute depletion of CTCF protein does not eliminate CTCF from all CBS, as thousands of sites remain occupied even after the removal of more than 90% of CTCF (Hyle *et al*, 2019; Luan *et al*, 2021). The stably bound CBS are, on one hand, depleted by the destabilizing downstream motif, and, on the other hand, enriched in the A/T‐rich motif located ~200 bp from the CTCF motif. Yet, the deletion of the A/T sequence does not affect CTCF binding as assessed at a silent *Myrip* locus. This suggests that additional factors are at play in the regulation of the stability of CTCF binding to chromatin (Luan *et al*, 2021). Promoters (Hyle *et al*, 2019) and enhancers (Luan *et al*, 2021) retain CTCF best, and a substantial fraction of CTCF peaks that withstand the degradation of the bulk of CTCF protein overlap TAD borders and loop anchors. Remarkably, the sites that do not lose CTCF in the auxin‐treated cells also remain occupied by CTCF during mitosis (Luan *et al*, 2021). More recent data has however challenged these observations showing that virtually all CTCF binding is lost from the CBS upon a 3‐h depletion of the CTCF protein (preprint: Hsieh *et al*, 2021). It will be essential to determine what regulates the stability of CTCF binding to TAD borders and loop anchors.

### RNA regulates CTCF binding to DNA

RNA modulates CTCF occupancy in the genome indirectly and directly. Local transcription of long non‐coding RNAs can induce nucleosome repositioning and occlusion of CBS thereby evicting CTCF (Lefevre *et al*, 2008). Furthermore, CTCF harbours an RNA‐binding region (RBR) and associates with RNA *in vivo* (Saldaña‐Meyer *et al*, 2014; Kung *et al*, 2015). The RNA species that interact with CTCF are frequently produced in the vicinity of CBS and can locally and directly impact the association of CTCF with DNA. RNA can stabilize CTCF binding to chromatin (Hansen *et al*, 2019; Saldaña‐Meyer *et al*, 2019) or disrupt it possibly by competing with DNA (Sun *et al*, 2013; Oh *et al*, 2021a). Global transcriptional inhibition blocks CTCF binding primarily at promoters, which suggests a positive role of RNA in CTCF binding at these locations (Hansen *et al*, 2019; Saldaña‐Meyer *et al*, 2019; Miyata *et al*, 2021). (See also below.)

## Factors controlling architectural functions of CTCF

There are tens of thousands of CBS in the genome, yet only a small fraction of them participates in the formation of TAD boundaries and loops. The architectural functions of CBS are likely a result of the regulatory element composition of TAD boundaries. Furthermore, local transcriptional activity may impinge on CTCF binding to chromatin. Likewise, the unique features of the CTCF protein including the ability of CTCF to interact with cohesins, the particularly stable binding of CTCF to chromatin, and the capacity of CTCF to homo‐oligomerize and form clusters are most probably essential for its architectural roles.

The unstructured N‐terminal tail of CTCF underlies the dialogue between CTCF and cohesins (Li *et al*, 2020; Nora *et al*, 2020; Pugacheva *et al*, 2020) and stabilizes cohesin binding to chromatin by directly competing with the cohesin release factor Wapl (Li *et al*, 2020). Remarkably, tethering of the unstructured CTCF termini to an unrelated ectopic genomic region using artificial ZFs is not sufficient to efficiently retain cohesin and block loop extrusion (Pugacheva *et al*, 2020), suggesting a combined role with other portions of the CTCF molecule in cohesin retention.

The non‐coding‐RNA steroid receptor RNA activator (SRA) and DEAD‐box RNA helicase p68 (DDX5) associate with CTCF at the IGF2/H19 locus. Knockdown of DDX5 and SRA hampers the association between CTCF and cohesins at the IGF2/H19 locus impacting allele‐specific expression patterns of Igf2/H19 (Yao *et al*, 2010). RNA stimulates the interactions between CTCF molecules *in vitro* (Yusufzai *et al*, 2004) and *in vivo* (Saldaña‐Meyer *et al*, 2014; Hansen *et al*, 2019). CTCF oligomerization in the cell nucleus is illustrated by a formation of clusters of variable sizes (Gu *et al*, 2020; Hansen *et al*, 2020). Deletion of the region in CTCF which is essential for CTCF–RNA interaction diminishes CTCF cluster size (Hansen *et al*, 2019), which is consistent with older data showing that a p53 antisense transcript Wrap53 favours CTCF clustering (Saldaña‐Meyer *et al*, 2014). Likewise, mutation of the RBR, precluding the association of CTCF with RNA, reduces CTCF binding at CBS and dismantles chromatin loops (Hansen *et al*, 2019; Saldaña‐Meyer *et al*, 2019). Noteworthy, RBR‐dependent cluster formation seems to aid CTCF in finding its cognate sites (Hansen *et al*, 2020), leading to the proposal that RNA molecules could act as road signs attracting CTCF and modulating its binding and perhaps also its architectural functions. Other data show that while this model can stand, the interactions between CTCF and RNA are complex and impact CTCF binding in a locus‐specific manner. Global transcription inhibition stabilizes CTCF clusters (Gu *et al*, 2020), and several non‐coding RNAs have been shown to destabilize the interaction between CTCF and DNA, which leads to loss of CTCF–CTCF loops (Sun *et al*, 2013; Oh *et al*, 2021a). Together, these data indicate that the non‐coding RNA portfolio in the cell might constitute an additional regulatory layer acting to fine tune CTCF binding to its cognate sites thereby impacting chromatin topology.

CTCF can undergo various post‐translational modifications, but their functional significance needs to be deepened further. Poly(ADP)ribosylation (PARylation) was shown to mark insulator‐bound CTCF proteins in low throughput chromatin immunoprecipitation coupled with microarray experiments (Yu *et al*, 2004). Treatment of cells with PARP1 inhibitor Olaparib attenuates insulator activity as measured by reporter assays. CTCF interacts with Poly‐ADP‐ribose polymerase I (PARP1) thereby impacting circadian gene repositioning to the heterochromatic nuclear lamina (Zhao *et al*, 2015). It is unclear how to combine these observations to a model that explains the contribution of PARP1 and PARylation in CTCF‐anchored loop and TAD formation. In addition to PARP1, there are numerous other proteins that co‐immunoprecipitate with CTCF (Uuskula‐Reimand *et al*, 2016). Amongst them, Myc‐Associated Zinc Finger Protein MAZ that was recently identified as a regulator of insulator functions of CTCF. CTCF‐ and MAZ‐binding sites coincide in the genome. The depletion of MAZ destabilizes CTCF‐anchored loops and diminishes insulation of PEI by CTCF genome‐wide (Xiao *et al*, 2021; Ortabozkoyun *et al*, 2022).

## CTCF‐mediated chromatin topology during development

The genomic coordinates of TADs are overall rather preserved in distinct cell types (Dixon *et al*, 2012; Rao *et al*, 2014); more domain boundaries are shared than cell type‐specific between largely transcriptionally divergent pluripotent and neural stem cells (Dixon *et al*, 2015; Bonev *et al*, 2017; Pekowska *et al*, 2018). There are several notable examples of loci that alter interaction patterns upon differentiation including, for instance, the Sox2 locus (Li *et al*, 2014) or the Hox loci (Montavon *et al*, 2011; de Laat & Duboule, 2013; Rodríguez‐Carballo *et al*, 2017). TAD boundaries and loops emerge early during development and increase their strengths stepwise during cell commitment accompanying loss of totipotency (Flyamer *et al*, 2017), exit from pluripotency and lineage commitment (Bonev *et al*, 2017; Pekowska *et al*, 2018). The consolidation of TADs detected by Hi‐C corresponds to the decrease in domain intermingling in super‐resolution microscopy experiments (Szabo *et al*, 2020). Chromatin topology featuring more loose TAD borders and infrequent loops is characteristic for ES cells. These features of nuclear structure can be reinstalled in differentiated cells upon reprogramming to pluripotency (Pekowska *et al*, 2018). Cell maturation also enhances architectural loop formation. Transcriptional activation at the globin genes in erythroid cells correlates with the strengthening of the CTCF–CTCF loop that demarcates the locus (Hua *et al*, 2021). Likewise, neuronal commitment of progenitor cells further consolidates TADs and loops (Bonev *et al*, 2017). Akin to cell differentiation, cell maturation is also accompanied by changes in CTCF‐mediated chromatin topology. Activation of naïve B cells leads to exit from the G0 phase and massive upregulation of gene expression. This phenomenon is associated with gain of CTCF–CTCF loops and induction of PEI (Kieffer‐Kwon *et al*, 2017). It is unclear whether loop and TAD boundary strengthening is related to differences in cohesin loading. In this light, the recent base pair resolution chromatin conformation capture experiments revealed that the increase in Nipbl binding correlates with transcriptional upregulation and loop strengthening at the activated loci suggesting that gain of loops may indeed be caused by increased cohesin loading upon activation of the locus (Hua *et al*, 2021).

What is the functional role of TAD consolidation during development? While this question is under investigation, CTCF seems to stabilize the acquired cell identity, its removal leads to increased spontaneous dedifferentiation of ES cells to totipotent‐like cells in cell culture (Olbrich *et al*, 2021; Zhu *et al*, 2021). Furthermore, removal of CTCF at the early stages of B‐cell to macrophage trans‐differentiation favours the transition (Stik *et al*, 2020). Therefore, chromatin structure might help in maintaining acquired cell identities.

## Implication of CTCF in disease

**Figure 3:**
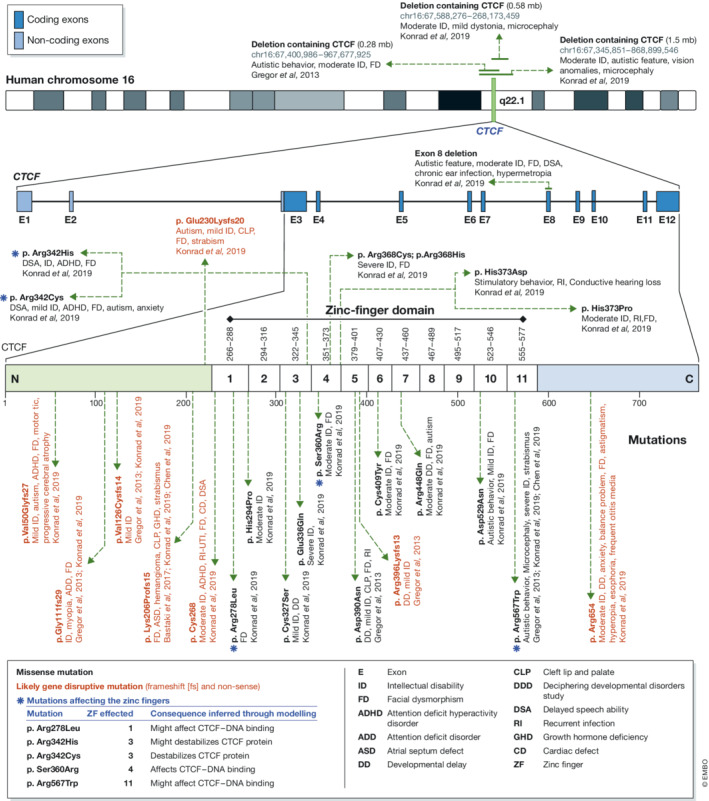
Mutations in CTCF related to neurological syndromes Multiple mutations including deletions have been reported for CTCF. These genetic perturbations are linked to numerous neurological manifestations. Genetic variants impacting CTCF binding sites associate with several disorders including neurological diseases. The predicted impact of the mutations in ZFs of CTCF on its 3D protein structure and the inferred possible effects on CTCF binding to chromatin.

Genomic rearrangements can reshuffle the relative positions and distances between regulatory elements leading to altered expression patterns underlying disease states. Loss of CBS and TAD boundary activity can result in severe phenotypical consequences including homeotic transformations (deletion of the boundaries at the HoxA and HoxC loci; Narendra *et al*, 2016), alteration in digit numbers when the boundaries at the WNT6/IHH/EPHA4/PAX3 are misplaced (Lupiáñez *et al*, 2015), or a plethora of phenotypes including cleft palate, delayed ossification, short snout and shortened long bones in the case of domain boundary inversions and ectopic insertions, which reshuffle promoter–enhancer contacts at the Sox9/Kcjn2 locus (Despang *et al*, 2019). Likewise, in addition to DNA mutations and epimutations in the CBS, altered protein sequences and expression levels of CTCF are related to several human disorders including cancer and neurological conditions (Fig 3). As we will see, the impact of CTCF is largely context dependent. However, common effects of truncating mutations and deletions of CTCF on cell proliferation reveal CTCF as a critical regulator of normal cell and organ homeostasis.

## Mutations of CTCF coding sequence in cancer and their functional role in oncogenesis

The DNA sequence encoding CTCF was first shown to be affected in breast and prostate tumours (Filippova *et al*, 1998). The analysis of several large Pan Cancer data sets including the TCGA data revealed a whole spectrum of CTCF mutations including deletions, amplifications and point mutations present in multiple tumours with varying frequency (Rubio‐Perez *et al*, 2015; Debaugny & Skok, 2020). Out of 273 detected mutations in CTCF (https://www.intogen.org/search?gene=CTCF accessed on the 19.03.2022), only 41 (15%) are synonymous while 165 are missense (60%) and 66 (24%) are truncating mutations; the genetic alterations localize throughout the coding sequence of CTCF and multiple mutations target the ZFs of CTCF (see also below; Rubio‐Perez *et al*, 2015).

CTCF acts as a tumour suppressor gene (Davoli *et al*, 2013; Gonzalez‐Perez *et al*, 2013; Rubio‐Perez *et al*, 2015). As we have noted above, most of the genetic alterations remove one allele of CTCF and were suggested to act as cancer drivers in breast, head and neck and uterine carcinomas (Gonzalez‐Perez *et al*, 2013) and endometrial cancers (Marshal *et al*, 2017). Prostate, ovarian and breast cancers frequently feature hemizygous deletions of CTCF (Filippova *et al*, 2002; Damaschke *et al*, 2020) and loss of one allele of CTCF in kidney and endometrial cancers correlates with poor patient survival (Kemp *et al*, 2014; Uhlen *et al*, 2017). What is the functional impact of the reduction of CTCF levels and how does CTCF prevent oncogenesis? Deletion of one copy of CTCF leads to loss of approximately 30% of CTCF protein in mouse embryonic fibroblasts (MEFs; Aitken *et al*, 2018). A diminished level of CTCF increases the susceptibility of the hemizygous animals to both spontaneous and radiation‐induced cancers (Kemp *et al*, 2014). The mechanism of the tumour suppressor action of CTCF might involve deregulated DNA methylation – the loss of CTCF binding can result in hypermethylation of CpGs (Kemp *et al*, 2014; Damaschke *et al*, 2020). Only a small fraction of up to a thousand CTCF peaks is lost in the Ctcf^+/−^ MEFs, yet these sites seem particularly relevant as judged by the fact that they predominantly intersect gene promoters (Aitken *et al*, 2018). Hemizygous deletion of CTCF leads to downregulation of both the mRNA and protein levels of multiple genes linked to oncogenic pathways (Aitken *et al*, 2018). The affected loci frequently contain multiple CBS and display an altered chromatin signature at their promoter regions including diminished looping to putative enhancer elements (Aitken *et al*, 2018). Remarkably, similar sets of genes are deregulated in Ctcf^+/−^ MEFs, spontaneously arising murine liver cancers as well as in human cancers with deleterious CTCF mutations (Aitken *et al*, 2018) which testifies the tumour suppressor action of CTCF. Further sustaining the view of a tumour suppressive action of CTCF, its loss leads to upregulation of the Programmed Cell Death Protein 1 (PD‐L1) which aids cancer cells to evade immune system surveillance (Martin *et al*, 2021; Oreskovic *et al*, 2022). Together, these data strongly argue for a tumour suppressive role of CTCF.

While the overexpression of CTCF blocks cell growth by slowing down the cell cycle (Rasko *et al*, 2001), some point mutations in CTCF that lead to gain of CTCF function can enhance cell survival by blocking apoptosis, as in the case of endometrial carcinomas (Marshal *et al*, 2017). Interestingly, adrenocortical carcinomas and testicular germ cell cancers frequently feature amplifications of the CTCF coding sequence (Debaugny & Skok, 2020), which might lead to CTCF overexpression. It will be important to determine whether and how the gain of CTCF might favour cancer development and whether the gain of CTCF copies constitutes a cause or a consequence of oncogenic pathway activation.

### CTCF‐binding sites in cancer

Numerous mutations and epimutations in CBS can contribute to transcriptional deregulation in diseases. Cancers frequently feature the A•T>C•G or A•T>G•C substitutions within the CBS or sequences immediately adjacent to it (Katainen *et al*, 2015; Kaiser *et al*, 2016; Poulos *et al*, 2016; Guo *et al*, 2018), which likely leads to loss of insulator sites. Loss of CBS may elicit aberrant gene expression. For instance, the deletion of CTCF‐ bound insulators can lead to upregulation of *TAL1* and *LMO2* proto‐oncogenes in T‐cell acute lymphoblastic leukemia (Hnisz *et al*, 2016). In human Gliomas, mutations in the Isocitrate Dehydrogenase (IDH) gene functionally limit the action of Tet enzymes and induce CpG island methylator phenotypes (G‐CIMP). The resulting epimutations lead to loss of CTCF binding and disruption of TAD boundaries (Flavahan *et al*, 2016) accompanied by aberrant gene expression as exemplified at the locus coding *PDGFRA*, a glioma oncogene. A similar effect is observed in gastrointestinal stromal tumors where hypermethylation of CTCF motifs within the CBS at domain boundaries at *KIT* and *FGF4* loci favours their expression (Flavahan *et al*, 2019).

It is unclear which insulator elements are most relevant in cancer. Cornell Non‐Coding Driver (CNCDriver) is a computational method that uses mutational imprints of distinct cancers to identify insulator drivers that affect the dialogue between promoters and enhancers thereby leading to cancer‐related gene deregulation (Liu *et al*, 2019). The cytokines of the TGFB family including *TGFB1* are involved in metastasis of multiple cancer types (Padua & Massagué, 2009). CNCDriver uncovered a mutation in the CTCF motif at the CBS in the vicinity of the *TGFB1* locus present in 17% of metastatic melanoma samples. This CBS might act as an insulator decreasing the expression of the *TGFB1* gene (Liu *et al*, 2019).

As we saw above, in murine B cell tumours, the translocation of the immunoglobulin heavy chain (IGH) locus to the vicinity of the oncogene *Myc* brings along the IGH enhancer. At the resulting chromosome, the IGH enhancer can aberrantly upregulate Myc expression (Gostissa *et al*, 2009). This translocation also includes a cluster of CBSs that flank the IGH enhancer. The deletion of these CBS has two effects: downregulation of Myc expression due to inefficient Myc‐IgH enhancer communication in the absence of these CBS (which correlates with decreased proliferation of the cancer cells) and an ectopic activation of genes otherwise separated from the IGH enhancer by the CBSs (Vian *et al*, 2018). Hence, CBS impact enhancer activity at rearranged chromatin loci in cancer.

Interestingly some cancer‐related point mutations in CTCF affect the individual ZFs of CTCF thereby altering its genomic binding profile (Filippova *et al*, 2002). The cancer‐related mutations L309P, R339Q and R377H affecting the ZF2, ZF3 and ZF4/5, respectively cause variable impact on CTCF binding to its cognate sites as assessed by ChIP (preprint: Bailey *et al*, 2021). Remarkably, the residue R377 is a hotspot of cancer mutations that may affect CTCF binding to DNA (preprint: Bailey *et al*, 2021). The R377 is frequently mutated in uterine, skin and bowel cancer (cancerhotspots.org, accessed 31.05.2022). Efforts are on the way to establish the functional implications of ZF mutations for cancer growth and development, using CRISPR‐Cas9 driven mutation and ChIP‐seq experiments.

## Role of CTCF in neuronal diseases

In humans, mutations in chromatin‐related factors often manifest themselves with neurological syndromes including intellectual impediments (Janowski *et al*, 2021). Patients with a heterozygous, four base pair deletion in the 5' end of the coding sequence of CTCF display developmental delay, intellectual disability and microcephaly (MIM#615502). This mutation shifts the reading frame of CTFC mRNA and introduces a premature stop codon thereby functionally knocking out one copy of the CTCF allele (Gregor *et al*, 2013). More recently, genetic analyses of 39 individuals with neurodevelopmental disorders (NDD) from mild to severe symptoms unveiled more CTCF variants related to neurological diseases (Konrad *et al*, 2019; Fig 3A) including mutations that might affect CTCF binding to DNA (Fig 3). Most of these variants reported in the study were *de novo* mutations, except for two types of familial CTCF mutations with mild effects of developmental delay.

Single Nucleotide Polymorphism (SNPs) at CBS have also been linked to neurological diseases (Table 1). The rs1990620 SNP is a risk variant linked to frontotemporal lobar degeneration, a complex disorder featuring a progressive decline in behaviour and dementia. The rs1990620 located upstream of the TSS of the transmembrane protein 106B (*TMEM106B*) gene increases CTCF binding. Overexpression of *TMEM106B* is neurotoxic and the genetic alteration correlates with a gain of chromatin contacts between the risk locus and the surrounding regulatory elements including the promoter of *TMEM106B* and a putative enhancer. This alteration of chromatin structure might lead to increased *TMEM106B* expression and decreased neuronal cell survival (Gallagher *et al*, 2017). Numerous other SNP risk variants of neurodegenerative diseases have been shown to intersect CBS (Gallagher *et al*, 2017) suggesting essential roles of CTCF in maintaining proper neuronal functions. Remarkably, schizophrenia disease risk variants often overlap anchors of CTCF–CTCF loops present in induced pluripotent stem cells‐derived neuronal cells (Rajarajan *et al*, 2018). These data suggest that changes in insulator activity contribute to transcriptional deregulation in this complex disease.

**Table 1:** Single nucleotide polymorphism (SNP) impacting CTCF functionality and disease susceptibility.

SNP	Locus	Functional impact	Disease	References
rs2535629	ITIH3 (3p21.1)	Disrupts CTCF binding and regulates the expression of the SFMBT1	Schizophrenia	Li *et al* (2022)
rs796364; rs281759	2q33.1	Disrupts CTCF, RAD21 and FOXP2 binding leading to upregulation of TYW5 (schizophrenia associated factor in brain)	Schizophrenia	Li *et al* (2022)
rs1990620	TMEM106B (7p21)	Increase in CTCF binding facilitates long range chromatin interactions perhaps leading to the upregulation of TMEM106B	Frontotemporal lobar degeneration	Gallagher *et al* (2017)
rs3825427	UBAC2 gene (13q32.3)	Increase in UBAC2 expression by recruiting CTCF at the promoter	Noise induced hearing loss	Wan *et al* (2022)
rs34481144	IFTIM3 (11p15.5)	Recruits CTCF to the promoter and downregulates the expression of IFTIM3	Influenza disease	Allen *et al* (2017)
rs9820407	CTNNB1 (3p22.1)	Increase in CTNNB1 expression possibly by CTCF mediated long range chromatin interaction	Osteoporosis	Wang *et al* (2021)

## Concluding remarks

CTCF is considered as one of the essential proteins implicated in the regulation of chromatin topology and gene expression. The recent data discussed here increase our understanding of the contribution of CTCF to the regulation of gene expression by directly acting at a promoter or by impacting promoter–enhancer contacts. Yet, a number of aspects of CTCF biology are still unclear (Box: In need of answers) and addressing these questions will be key to link chromatin topology to gene expression during development and in disease. Activation of regulatory elements increases their mobility (Gu *et al*, 2018) and association with mediator condensates (Cho *et al*, 2018b), which is attenuated when transcription is inhibited (Gu *et al*, 2018). How can one reconcile these observations? Gene activation correlates with a gain in intra‐TAD interactions (Kieffer‐Kwon *et al*, 2017; Pekowska *et al*, 2018) at least in part due to increased cohesin loading at activated promoters and enhancers (Hua *et al*, 2021). At sites where chromatin‐bound CTCF flanks an enhancer (Fig 1C), PEI are strengthened by CTCF and form architectural stripes (Vian *et al*, 2018). Recent nanoscopy and scanning electron microscopy data revealed that CTCF and active chromatin marks, including histone modifications typical for active enhancers, co‐localize on the surface of chromatin domains reminiscent of TADs (Miron *et al*, 2020). It is therefore possible that at loci forming architectural stripes, the enhancer is confined to the TAD surface and by fixing its position in space the PEI may be formed more efficiently.

The frequently observed, relatively subtle transcriptional effects accompanying TAD–TAD merging in the genome engineering experiments agree with the overall weak effect of cohesin and CTCF removal on gene expression (Nora *et al*, 2017; Rao *et al*, 2017). These data might be confounded by the fact that the gene expression redout comes from a population of cells. CBS contribute to the cell‐to‐cell variability in gene expression (Ren *et al*, 2017) particularly affecting transcriptional regulators implicated in the maintenance of cell identity (Wang *et al*, 2019). Corollary to this, the transcriptional effects of CTCF disruption might require time to fully manifest. Indeed, disruptions of individual CBS often perturb gene expression in long term.

Several groups described widespread immunity of promoters to the influence of new enhancers within the context of reshuffled TADs built at loci that underwent genomic rearrangements (Despang *et al*, 2019; Ghavi‐Helm *et al*, 2019; Laugsch *et al*, 2019). What else, apart from insulators, affects the responsiveness of promoters to enhancers? The sequence composition of regulatory elements likely plays an important role in this process (Zabidi *et al*, 2015; Arnold *et al*, 2017; Pachano *et al*, 2021). Furthermore, the combinatorial cis‐regulatory element landscape of each TAD impacts the promoter–enhancer dialogue by creating a competitive environment actively shaping the likelihood of establishing functional links between genes and enhancers (Lower *et al*, 2009; Furlong & Levine, 2018; Cho *et al*, 2018a; Hao *et al*, 2019; Oudelaar *et al*, 2019; Oh *et al*, 2021b; Zuin *et al*, 2022). TADs are in fact highly dynamic structures constantly built by cohesins and dismantled by the Wapl cohesin unloader (Haarhuis *et al*, 2017; Hansen *et al*, 2017; Bintu *et al*, 2018; Vian *et al*, 2018). Recent live‐cell imaging of loop dynamics in ES cells revealed that CTCF–CTCF loops are rather rare (3–6% of loci form loops at any given time in the cell population) and persist for up to 30 min (Gabriele *et al*, 2022). Depending on the composition of the cohesin complex, loops may have varying lifetimes (Wutz *et al*, 2020). It will be important to understand what regulates the composition of the cohesin complexes at a given genomic locus, how the cohesin composition impacts CTCF functions and whether it affects PEI. Likewise, understanding the mechanisms by which CTCF regulates locus‐specific gene expression will illuminate underlying mechanisms of neurological disorders and likely help to increase our understanding of oncogenesis.

## Author contributions

**Bondita Dehingia:** Writing – original draft; writing – review and editing; visualization. **Małgorzata Milewska:** Writing – original draft; writing – review and editing; visualization. **Marcin Janowski:** Writing – original draft. **Aleksandra Pękowska:** Conceptualization; resources; supervision; funding acquisition; visualization; writing – original draft; project administration; writing – review and editing.

In addition to the CRediT author contributions listed above, the contributions in detail are:

AP – conceptualization. All the authors contributed to manuscript writing.

## Disclosure and competing interests statement

The authors declare that they have no conflict of interest.
